# Preparations of 25 wt% of Pyraclostrobin Nanosuspension Concentrate (SC) Using Lignosulfonate-Based Colloidal Spheres to Improve Its Thermal Storage Stability

**DOI:** 10.3390/molecules29071419

**Published:** 2024-03-22

**Authors:** Qianqian Tang, Yu Sun, Jinnuo Li, Mingsong Zhou, Dongjie Yang, Yuxia Pang

**Affiliations:** 1 State Key Laboratory of Pulp and Paper Engineering, School of Chemistry and Chemical Engineering, South China University of Technology, 381 Wushan Road, Tianhe District, Guangzhou 510640, China; lscxcyjy@163.com (Y.S.); cedjyang@scut.edu.cn (D.Y.); ceyxpang@scut.edu.cn (Y.P.); 2 Henan Key Laboratory of Function-Oriented Porous Materials, College of Chemistry and Chemical Engineering, Luoyang Normal University, 6 Jiqing Road, Yibin District, Luoyang 471934, China; lhltqq1987@163.com (Q.T.); 17333740259@163.com (J.L.)

**Keywords:** lignosulfonate-based colloidal spheres, polycarboxylate dispersants, pyraclostrobin nanosuspension concentrate

## Abstract

Improving the thermal storage stability of nanosuspension concentrate (SC) prepared from low-melting-point pesticide is a recognized problem. In this work, using pyraclostrobin as the raw material, 25 wt% of pyraclostrobin nano-SC was prepared through a water-based grinding method, and the optimal grinding conditions were obtained as follows: a grinding time of 23 h, D-3911 as dispersant and a dispersant dosage of 12 wt%. The pyraclostrobin nano-SC D_90_ size prepared based on this best formula was 216 nm. Adding glycerin could improve the stability of nano-SC at room temperature, but its thermal storage stability was still poor. For this problem, sodium lignosulfonate and cetyltrimethylammonium bromide (NaLS/CTAB) colloidal spheres were prepared through electrostatic and hydrophobic self-assembly and characterized. The delamination and precipitation of nano-SC can be significantly improved by adding an appropriate amount of colloidal spheres, and the nano-SC D_90_ size decreased from 2726 to 1023 nm after 7 days of thermal storage. Farmland experiments indicated the control efficiency of pyraclostrobin nano-SC against flowering cabbage downy mildew disease was about 30% higher than that of SC. Especially after adding the wetting agent, the effect of nano-SC could be comparable to that of commercial Kairun (currently the best pyraclostrobin formulation in the world).

## 1. Introduction

At present, the population of the world is increasing every day, with a corresponding increase in food demand [1], so providing sustainable food production for a rapidly growing population is one of the major challenges facing the global agricultural sector. Increasing the use of pesticides and fertilizers has become necessary to maximize agricultural productivity [2]. According to statistics, the current global annual use of pesticide formulation has exceeded 4.6 million tons [3]. Although pesticides play a beneficial role in agriculture, they can be harmful to humans and other non-target organisms depending on their toxicity, contamination level and exposure duration. Their use (overuse) also creates resistance in pests, affects food quality and generates significant waste [4]. In addition, it is estimated that more than 90% of pesticides is lost to the environment during the application stage or resides in agricultural products, which not only causes pollution to the environment [3] but also raises the application cost for farmers. Therefore, safe and efficient pesticide application methods are essential to prevent the adverse effects of pesticides. In this direction, nanotechnology offers great promise and can be used as an innovative tool for the safe delivery of agrochemicals. To apply nanotechnology in the pesticide field can significantly reduce the pesticide dosage and improve the pesticide effect [5]. Due to the development prospects of nanotechnology in the pesticide field, the International Union of Pure and Applied Chemistry (IUPAC) ranked nanopesticides as number one in the top ten emerging chemical technologies that would change the world in 2019 [6].

Until now, there has been no unified definition regarding nanopesticides, and pesticide preparations with a particle size lower than 1000 nm or new characteristics associated with a small size are generally widely defined as nanopesticides [7]. Pesticide nanosuspension concentrate (SC) is a nanopesticide that is obtained by dispersing the insoluble pesticide into water with the aid of a dispersant through nanotechnology [8]. Nano-SC can enhance the permeability and adhesion percentage of a pesticide on leaf surfaces, extend its effective period, reduce its residual pollution, but also improve its biological activity, so as to reduce its dosage and increase the crop yield, improving the overall economic benefits [9]. There are many methods for preparing nano-SC, which boil down to two types, namely the top-down method [10,11,12,13] and the bottom-up method [14,15,16]. Among them, the water-based grinding method, a top-down method, has advantages such as a simple preparation technology, low costs, being environmentally friendly and easy to realize industrialization [17], and therefore is a simple and suitable way to prepare nano-SC with insoluble insecticides.

In recent years, pyraclostrobin has been in the world’s top two sales of fungicide. It has a low water solubility (only 1.9 mg/L) and a low melting point (63.7–65.2 °C), which can inhibit the mitochondrial respiration of fungal cells, resulting in the fungus being unable to obtain energy and eventually dying. Pyraclostrobin has the highest activity of methoxyacrylate fungicides, and mainly has two formulations of SC and emulsifiable concentrate (EC), of which EC contains a toxic organic solvent, and has disadvantages of environmental pollution, unsafe storage and transportation and easily causes drug damage, etc. At present, there is little research on pyraclostrobin nano-SC, and it is very important to prepare pyraclostrobin nano-SC to improve its pesticide effect. However, pyraclostrobin has a low melting point, which softens during grinding and thermal storage. These softened particles are more likely to stick together after colliding with each other, so the water-based preparation of pyraclostrobin is prone to agglomeration and delamination (phase separation) during production and storage and has a very poor thermal storage stability. Therefore, developing pyraclostrobin nano-SC has always been a difficult problem in the pesticide preparation field [18,19].

As one of the most abundant natural biomass resources [20,21,22,23,24], lignin is also a unique renewable aromatic polymer on Earth [25,26,27,28,29] that has been attracting increasing worldwide attention due to the more and more serious environmental pollution and oil resource crisis. Lignosulfonate (LS) is an important and typical derivative of lignin produced from the sulfite pulping process, and is often considered as an amphiphilic polymer, which contains both hydrophilic phenolic hydroxyl, sulfonic and carboxyl groups and hydrophobic aliphatic and aromatic groups [30,31]. This makes LS able to be transformed into colloidal spheres through self-assembly. Due to the rigidity, these prepared lignosulfonate-based colloidal spheres do not agglomerate due to collision, and, when they are added to pesticide nano-SC, these colloidal spheres collide with pesticide particles, and are expected to reduce the collision and agglomeration between pesticide nanoparticles themselves, thus enhancing the stability of the entire disperse system.

In this paper, several pyraclostrobin nano-SC systems were first prepared by the water-based grinding method, and the best grinding formula was determined. Afterwards, the structure of pyraclostrobin nano-SC prepared according to the best formula was characterized by laser particle size analyzer, scanning electron microscope (SEM) and X-ray diffractometer (XRD). Then, the effects of glycerin on the rheological performance, storage stability of nano-SC at room temperature and 54 °C (thermal storage stability) were studied. Next, using sodium lignosulfonate (NaLS) as a raw material, NaLS/cetyltrimethylammonium bromide (CTAB) colloidal spheres were prepared through electrostatic and hydrophobic self-assembly and then characterized by SEM and transmission electron microscopy (TEM). Subsequently, these prepared colloidal spheres were applied to improve the thermal storage stability of pyraclostrobin nano-SC. Finally, the pesticide effect of pyraclostrobin nano-SC against flowering cabbage downy mildew disease was tested through farmland experiments. This work developed a new pyraclostrobin nano-SC with good performance and provided technical support for preparing a pesticide nano-SC with a low melting point.

## 2. Results and Discussion

### 2.1. Determination of the Optimum Grinding Conditions of Pyraclostrobin Nano-SC

**Figure 1:**
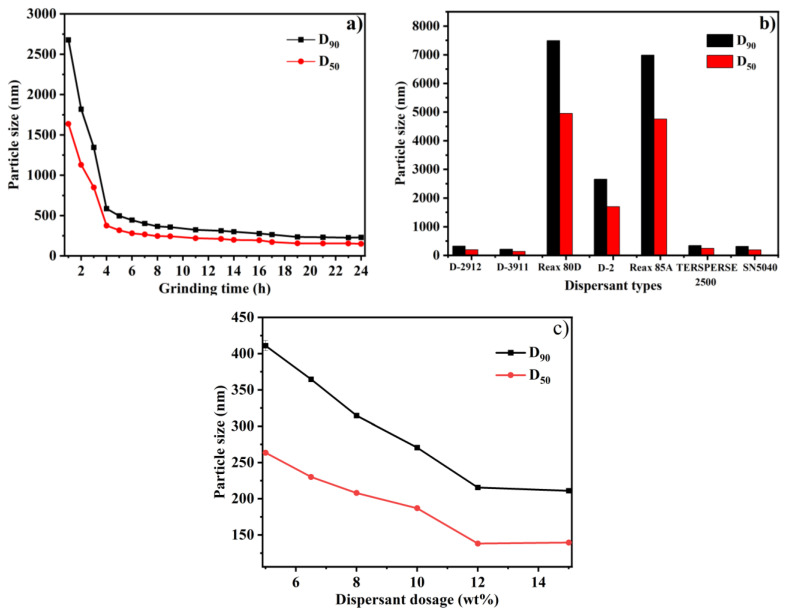
Effects of grinding time (**a**), dispersant types (**b**) and dispersant dosages (**c**) on D_90_ particle size of pyraclostrobin nano-SC.

Firstly, the effect of grinding time was studied. Using D-3911 as a dispersant and fixing the mass ratio of pesticide to zirconium dioxide balls at 1:3, several pyraclostrobin nano-SC systems with an effective pesticide content of 25 wt% were prepared according to the formula in Table S1 by controlling different grinding time, and their D_90_ particle size (the corresponding particle size when the cumulative particle size distribution number of a sample reached 90%) was separately determined, as shown in Figure 1a. It can be seen from Figure 1a that the nano-SC D_90_ particle size gradually decreased with the increasing grinding time, and reached a minimum 226 nm when ground for 23 h. Afterwards, the D_90_ particle size instead slightly increased. Therefore, the best grinding time was determined to be 23 h. During the grinding process, there was friction between the zirconium dioxide balls and the materials and zirconium dioxide balls under high shear forces, producing a huge amount of heat. For pyraclostrobin with a low melting point, a higher temperature caused the original pesticide particles to soften, so the particles were more likely to stick together, thus reducing the grinding efficiency. Therefore, grinding the pyraclostrobin particles to a nanometer level required more time.

Secondly, the effect of dispersant types was discussed. The mass ratio of pesticide to zirconium dioxide balls was still kept at 1:3 and the grinding time was fixed at 23 h. D-2912, D-3911, D-2, SN-5040, TERSPERSE 2500, Reax 85A and Reax 80D were separately selected as dispersants to prepare 25 wt% of the pyraclostrobin nano-SC system. The D_90_ particle size was then measured and the result is exhibited in Figure 1b. As shown in Figure 1b, the nano-SC D_90_ particle sizes prepared by lignin dispersants Reax 85A and Reax 80D were all above 5000 nm, indicating that the grinding efficiency of lignin dispersants on pyraclostrobin was very low and they were not suitable for preparing pyraclostrobin nano-SC. Among all the dispersant systems, the pyraclostrobin nano-SC D_90_ particle size prepared by D-3911 was the smallest, and was as low as 216 nm. So, D-3911 was selected as the best dispersant.

Finally, the effect of dispersant dosage was considered. In this section, D-3911 was utilized as the dispersant, and the mass ratio of pesticide to zirconium dioxide balls and the grinding time remained unchanged and were still fixed at 1:3 and 23 h, respectively. Then, the D_90_ particle size of 25 wt% of pyraclostrobin nano-SC systems prepared according to this grinding condition by changing different dispersant dosages was determined and is displayed in Figure 1c. It was found that the nano-SC D_90_ particle size gradually decreased with the increasing dispersant dosage and reached as low as 216 nm when the dispersant dosage was 12 wt%. At a dispersant dosage of 15 wt%, the nano-SC D_90_ particle size was smaller, only 211 nm, but there was a layer of micro-foam on the top after grinding. Therefore, the optimum dispersant dosage was determined to be 12 wt%.

According to the above-mentioned experimental results, the optimum grinding conditions were obtained as follows: a grinding time of 23 h, with D-3911 as the dispersant and a dispersant dosage of 12 wt%. Based on this optimum grinding condition and fixing the mass ratio of pesticide to zirconium dioxide balls at 1:3, 25 wt% of pyraclostrobin nano-SC was prepared and used for the following study.

### 2.2. Characterizations and Performance of Pyraclostrobin Nano-SC

**Figure 2:**
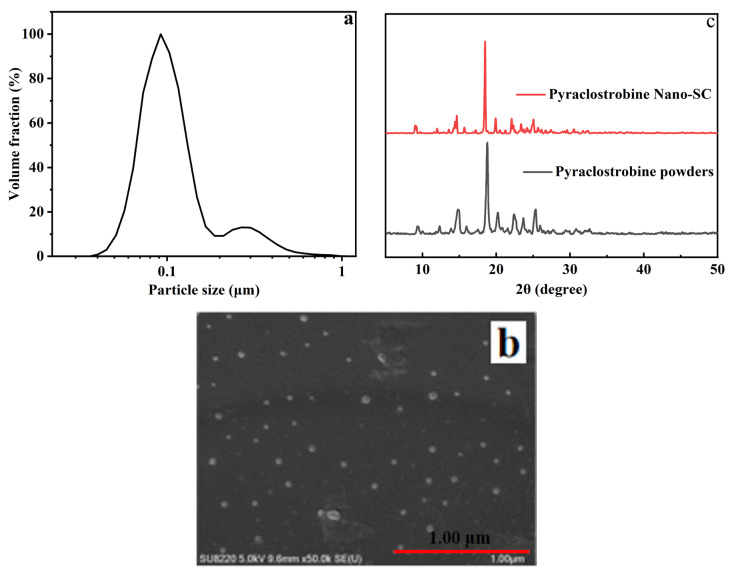
Particle size distribution (**a**), SEM image (**b**) of pyraclostrobin nano-SC (prepared according to the optimum grinding conditions) and X-ray diffraction spectrogram (**c**) of pyraclostrobin original pesticide powders and pyraclostrobin nano-SC.

Figure 2a,b show the particle size distribution and SEM image of pyraclostrobin nano-SC, respectively, prepared according to the optimum grinding conditions. It can be seen in Figure 2a that the pyraclostrobin nano-SC particle size was approximately 92 nm, and the appearance of double peaks may have been caused by the uneven grinding of pesticide particles. As shown in Figure 2b, the pyraclostrobin nano-SC particles showed a near spherical shape, and the particle size was generally below 100 nm, which was basically consistent with Figure 2a.

The crystal from of particles could affect the solubility, stability and bioavailability of nano-SC, which may have been changed due to the collisions between particles during the grinding process. Therefore, the XRD spectrograms of pyraclostrobin original pesticide powders and pyraclostrobin nano-SC were measured to explore the effect of grinding on the crystal shape of pyraclostrobin, as shown in Figure 2c. It was found that pyraclostrobin nano-SC showed three characteristic diffraction peaks at 18°, 22° and 25° (2θ), which were nearly consistent with the characteristic peaks of pyraclostrobin original pesticide powders. This indicated that the main crystalline structures of pesticide particles in pyraclostrobin nano-SC were maintained, but also some other crystalline structures were transformed. The amorphous structure can increase the solubility of insoluble pesticide particles. Therefore, to prepare pyraclostrobin original pesticide into nano-SC by grinding can increase its solubility in water.

### 2.3. Effects of Glycerin on the Properties of Pyraclostrobin Nano-SC

**Figure 3:**
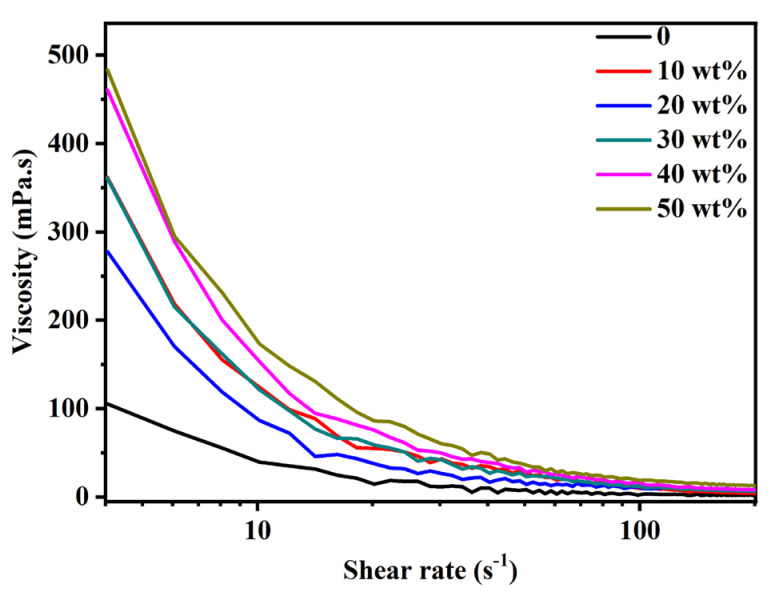
Viscosity/shear rate of pyraclostrobin nano-SC with different glycerin additive amount.

Firstly, the effects of glycerin on the rheological properties of pyraclostrobin nano-SC were determined, as shown in Figure 3. As can be seen in Figure 3, the apparent viscosity of nano-SC systems with different glycerin additive amounts (0, 10 wt%, 20 wt%, 30 wt%, 40 wt% and 50 wt%) all gradually decreased as the shear rate increased, showing a pseudoplastic fluid characteristic (shear thinning). Additionally, as the glycerin additive amount increased, the viscosity of pyraclostrobin nano-SC showed a gradually increasing trend. After the glycerin containing three hydroxyl groups was added, the hydrogen bond interaction between glycerin molecules and water and glycerin molecules was strengthened, resulting in the formation of a dynamic network structure. This mild and reversible dynamic network structure was beneficial to increasing the viscosity of pyraclostrobin nano-SC, weakening the Brownian movement of particles and inhibiting the sedimentation, thus improving the static stability of the system. As the glycerin additive amount of this dynamic network structure was strengthened, the viscosity of pyraclostrobin nano-SC further increased. Accordingly, the static stability of the system also improved.

**Figure 4:**
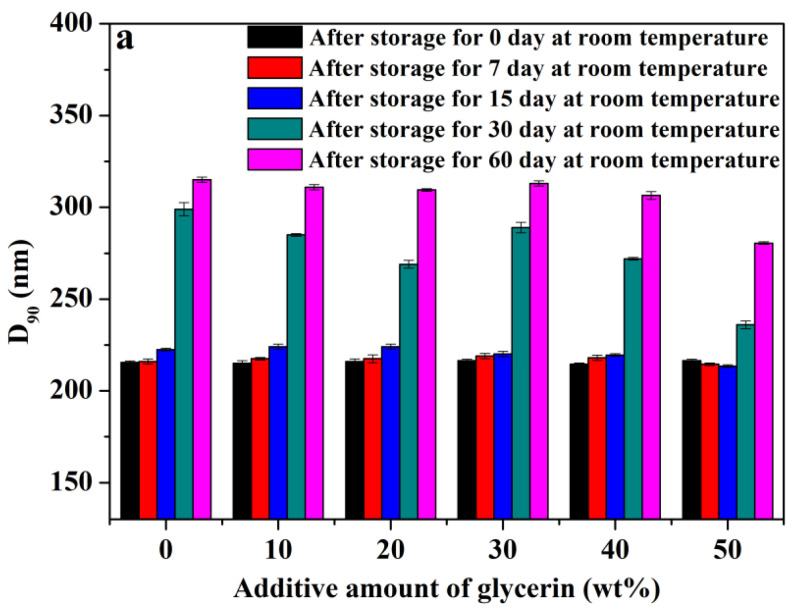

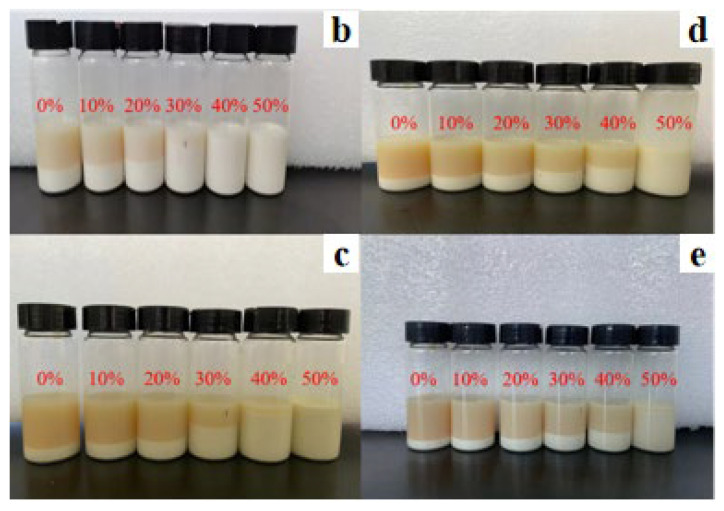
D_90_ particle size after storage separately for 0, 7, 15, 30 and 60 days at room temperature (**a**) and physical appearance after storage separately for 7 (**b**), 15 (**c**), 30 (**d**) and 60 (**e**) days at room temperature of pyraclostrobin nano-SC with different glycerin additive amount.

Secondly, to explore the effect of glycerin on the room temperature stability of pyraclostrobin nano-SC, 10 wt%, 20 wt%, 30 wt%, 40 wt% and 50 wt% of glycerin were separately added to nano-SC, which were then placed at room temperature for 0, 7, 15, 30 and 60 days, respectively. Subsequently, the D_90_ particle size and physical appearance of nano-SC were measured and recorded, as shown in Figure 4.

As shown in Figure 4a–e, the uniformity of all nano-SC systems was significantly improved with the increasing glycerin addition. Especially, when the glycerin addition amount was 50 wt%, only microprecipitation and slight delamination occurred even after nano-SC was placed at room temperature for 60 days, and the increment of D_90_ particle size was also smaller than that of nano-SC without glycerin. This indicated that glycerin could improve the stability of pyraclostrobin nano-SC at room temperature, and the addition of glycerin was more beneficial to the storage of nano-SC at room temperature.

**Figure 5:**
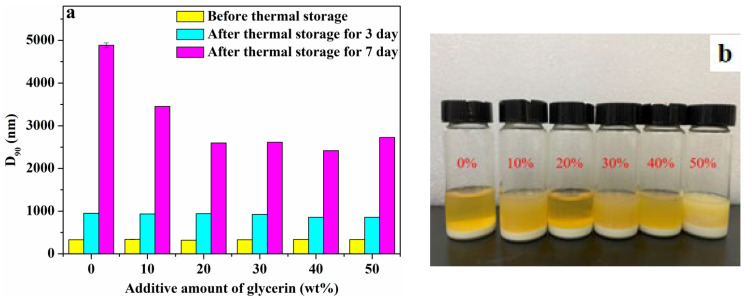
D_90_ particle size before and after thermal storage separately for 3 and 7 days (**a**) and physical appearance after thermal storage for 3 days (**b**) of pyraclostrobin nano-SC with different glycerin additive amounts.

Next, the effect of glycerin on thermal storage stability of pyraclostrobin nano-SC was investigated. Similarly, 10 wt%, 20 wt%, 30 wt%, 40 wt% and 50 wt% of glycerin was separately added to nano-SC, and then stored in an oven at 54 ± 1 °C for 3 and 7 days, respectively. Afterwards, the D_90_ particle size and physical appearance of nano-SC (after thermal storage for 3 days) were tested and recorded, as shown in Figure 5. As shown in Figure 5a, the D_90_ particle size of all systems showed an obviously increased trend after thermal storage, and the longer the thermal storage time, the larger the D_90_ particle size increment. In the thermal storage process, as the system temperature rose, the Brownian movement and thermal movement of particles were intensified, which led to the increased kinetic energy of particles. The collision intensity and frequency among particles also increased. In this case, agglomeration occurred easily among particles, and the particle size increased, as illustrated in Figure S1.

Additionally, before adding glycerin, the nano-SC D_90_ particle size after thermal storage for 7 days could increase to 4900 nm. But, after glycerin was added, the nano-SC D_90_ particle size after thermal storage for 7 days was smaller than 3500 nm, indicating that glycerin could enhance the thermal storage stability of nano-SC. The reason was analyzed as follows. The addition of glycerin could slightly enhance the viscosity of the system, and also a weak dynamic network structure was formed in the glycerin/water medium due to the hydrogen bonding interaction, which would both block the Brownian movement and thermal movement of particles to a certain extent and reduce the collision kinetic energy among particles. Therefore, the addition of glycerin could weaken the agglomeration among particles in the system to a certain extent, so that the growth of particle size was slightly moderated. Even so, the nano-SC D_90_ particle size after thermal storage for 3 days reached the micron level and there was obvious precipitation in the systems (Figure 5a,b). Therefore, pyraclostrobin nano-SC had difficulty achieving thermal storage stability. It was inferred that the melting point of pyraclostrobin was lower (63.7–65.2 °C), and the softening phenomenon occurred during thermal storage. The solid elastic collision between original pesticide particles became the inelastic collision of semi-solid substances, which made the particles more easily agglomerate with each other, resulting in a larger particle size and then a delamination. Furthermore, the effect of temperature was also a key factor. To be specific, as the temperature rose, the hydrophilicity of the D-3911 dispersant tended to increase, which was beneficial to its molecular stretch in solutions. In this case, it was easier for D-3911 to achieve a firm multi-point adsorption on pyraclostrobin particle surfaces. This was favorable for the physical stability of pyraclostrobin nano-SC. However, the increase in temperature would also intensify the Brownian movement of the pyraclostrobin nano-SC system. The collision kinetic energy of particles sharply increased. The violent collision among particles could make the adsorbed D-3911 dispersant fall off again, resulting in the reduction of the charge amount on the particle surface and the weakening of steric hindrance. This would lead to the agglomeration of nanoparticles during Brownian movement collision, causing the particle size to grow to a micron level. The nano-SC system would loss its kinetic stability to precipitate. In summary, the increase in temperature was also not conducive to the stability of pyraclostrobin nano-SC. Both these two factors led to the poor thermal storage stability of nano-SC prepared from low-melting-point pyraclostrobin.

### 2.4. Characterizations of NaLS/CTAB Colloidal Spheres and Their Effects on Thermal Storage Stability of Pyraclostrobin Nano-SC

**Figure 6:**
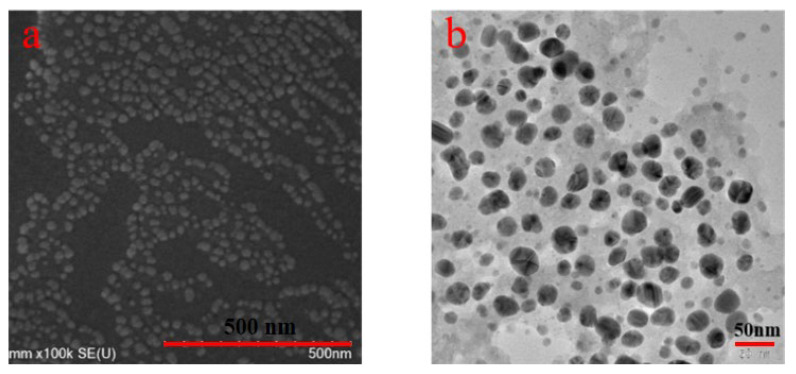
SEM (**a**) and TEM (**b**) images of NaLS-CTAB colloidal spheres.

Figure 6a,b show the SEM and TEM image of colloidal spheres, respectively. It can be observed that NaLS/CTAB colloidal spheres showed a spherical conformation. They were relatively uniform in size and most microspheres had a particle size of 20–30 nm.

Due to the low melting point of pyraclostrobin, the original pesticide particles softened very easily during thermal storage, and they could agglomerate under collision, leading to a larger particle size and then precipitation and delamination. Additionally, the increase in temperature was also not favorable for the stability of the nano-disperse system. Therefore, to improve the thermal storage stability of low-melting-point pyraclostrobin nano-SC was a difficult problem and challenge. In this study, we tried to utilize NaLS/CTAB colloidal spheres by synergy with glycerin to increase the thermal storage stability of pyraclostrobin nano-SC.

**Figure 7:**
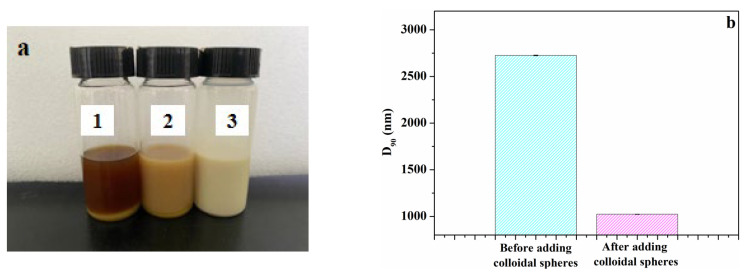
Physical appearance of pyraclostrobin nano-SC after thermal storage for 7 days (sample 1: containing 40 wt% of glycerin and 10 wt% of NaLS/CTAB colloidal spheres; sample 2: containing 45 wt% of glycerin and 5 wt% of NaLS/CTAB colloidal spheres; sample 3: containing 49 wt% of glycerin and 1 wt% of NaLS/CTAB colloidal spheres) (**a**) and pyraclostrobin nano-SC D_90_ particle size with 49 wt% of glycerin (after thermal storage for 7 days) before and after adding 1 wt% of colloidal spheres (**b**).

Different amounts of NaLS/CTAB colloidal spheres and glycerin were added into pyraclostrobin nano-SC and stirred well, followed by being placed in an oven at 54 ± 1 °C for thermal storage. The physical appearance of nano-SC after thermal storage of 7 days is displayed in Figure 7a. As can be seen from Figure 7a, a layer of precipitate appeared on the bottom of both suspension 1 and 2 after 7 days of thermal storage, but the precipitation amount was less than that of nano-SC without NaLS/CTAB colloidal spheres. In suspension 3, there was no stratification or precipitation. These experimental results indicated that the addition of NaLS/CTAB colloidal spheres could improve the thermal storage stability of pyraclostrobin nano-SC, and the effect was best when the additive amount of colloidal spheres was 1 wt%.

**Figure 8:**
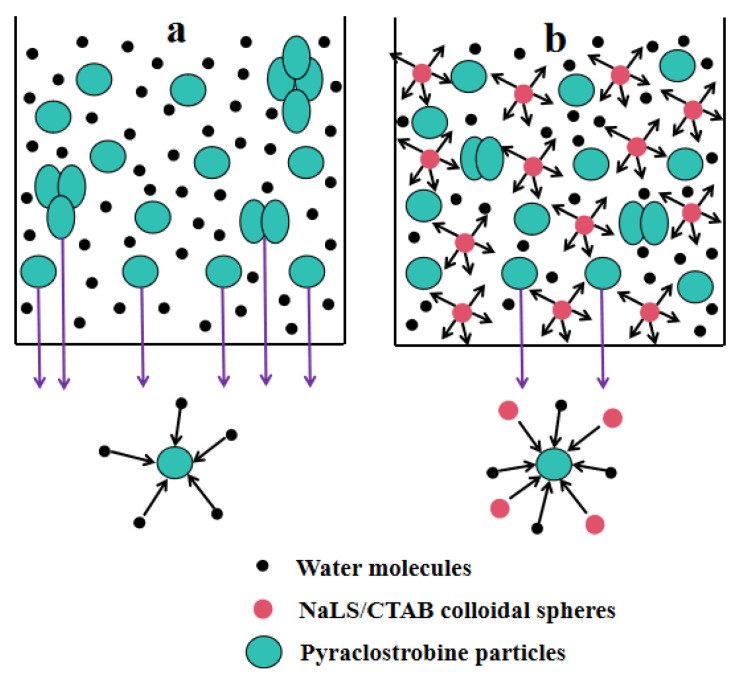
Interaction mechanism of NaLS/CTAB colloidal spheres for improving the storage stability of pyraclostrobin nano-SC ((**a**) before adding NaLS/CTAB colloidal spheres; (**b**) after adding NaLS/CTAB colloidal spheres).

The interaction mechanism of NaLS/CTAB colloidal spheres for improving the thermal storage stability of pyraclostrobin nano-SC is illustrated in Figure 8. The agglomeration of original pyraclostrobin particles in nano-SC can be explained from the kinetic aspect. Before adding NaLS/CTAB colloidal spheres (Figure 8a), pyraclostrobin nanoparticles spontaneously settled in nano-SC. Pyraclostrobin nano-SC was a polydisperse system, in which the collision among water molecules would cause the Brownian movement (a random movement in any direction) of pyraclostrobin nanoparticles. These nanoparticles mainly depended on Brownian movement to counter the gravity settling. But the Brownian movement was relatively weaker in case a, and the original pyraclostrobin particles were more likely to settle. During thermal storage, the Brownian movement of pyraclostrobin particles was more intense, which increased the collision probability among themselves. For original pyraclostrobin with a low melting point, the particles softened easily during thermal storage, and then adhered together when colliding. This led to more and more pyraclostrobin particles agglomerating and finally settling, destroying the stability of the entire disperse system. After adding NaLS/CTAB colloidal spheres (Figure 8b), the collision between pyraclostrobin particles was weakened. Because NaLS/CTAB colloidal spheres were solid with a small particle size (20–30 nm, as shown in Figure 6) and of a large quantity, they were filled with pyraclostrobin particles that played a blocking role, resulting in increased collisions between pyraclostrobin particles and colloidal spheres but reduced collisions between pyraclostrobin particles. Additionally, the non-directional Brownian movement also occurred in NaLS/CTAB colloidal spheres due to the impact of water molecules, and would drive these spheres to impact pyraclostrobin nanoparticles. Because NaLS/CTAB colloidal spheres were rigid and negatively charged [20], they would not agglomerate after colliding with themselves or other softened pyraclostrobin nanoparticles. Meanwhile, because this impact force was much larger than that of water molecules, pyraclostrobin particles would experience more intense Brownian movement, which could weaken the settlement trend of pyraclostrobin particles to a certain extent and increase the kinetic stability of nano-SC.

Although no stratification or precipitation appeared in suspension 3, the D_90_ particle size increased to 1023 nm (Figure 7b). It was assumed that the addition of an appropriate amount of NaLS/CTAB colloidal spheres could reduce the collision between original pesticide particles, but the softening phenomenon could not be avoided, and the particles still stuck to each other, finally resulting in an increased particle size of nano-SC. In summary, adding an appropriate amount of NaLS/CTAB colloidal spheres could improve the thermal storage stability of pyraclostrobin nano-SC, which not only solved the stratification and precipitation problem of nano-SC, but also inhibited the growth of particle size. The D_90_ particle size of suspension 3 with colloidal spheres was 1023 nm, but 2726 nm when no colloidal spheres were added, as shown in Figure 7b. However, due to the low melting point of pyraclostrobin, the pesticide particles softened and stuck to each other during thermal storage, which greatly limited the effect of NaLS/CTAB colloidal spheres.

### 2.5. Pesticide Efficiency of Pyraclostrobin Nano-SC on Flowering Cabbage Downy Mildew Disease

In this section, 25 wt% of pyraclostrobin nano-SC prepared based on the best grinding formula was used for the pesticide efficacy test on the prevention and treatment of flowering cabbage downy mildew disease, which was then compared with that of 25 wt% of pyraclostrobin SC and 25 wt% of pyraclostrobin EC (the EC product “Kairun” provided by BASF in Germany). The testing conditions are listed in Table S2. The quality improvement and yield increase effect of different pyraclostrobin preparations on flowering cabbage after application for 3 days are shown in Table 1.

It can be seen in Table 1 that the control efficiency of pyraclostrobin nano-SC against flowering cabbage downy mildew disease increased by about 30% compared with that of pyraclostrobin SC. Meanwhile, the leaves of flowering cabbage using nano-SC were darker green, with smaller leaf corners and better growth, and the plant height increased by about 6–10%, as shown in Figure S2. In addition, a small part of the leaf edge of the flowering cabbage in sample 5-2 was damaged by pesticide, but no such drug damage phenomenon was found in sample 5-1 (Figure S2), which indicated that there was a certain drug damage when the dosage of EC was relatively larger. Moreover, after adding the wetting agent sodium dodecyl benzenesulfonate, the control efficiency of pyraclostrobin nano-SC against flowering cabbage downy mildew disease improved by 30–50%, and the plant height of flowering cabbage also obviously increased, but the control efficiency of pyraclostrobin SC after adding the wetting agent barely improved (Table 1).

Among all pesticide formulations, EC was recognized as one of the most effective. Kairun is currently the best product in the world among various preparations with the same pyraclostrobin content. As shown in Table 1, compared with SC, the control efficiency of Kairun against flowering cabbage downy mildew disease improved by nearly three times, and Kairun showed a more excellent effect on the plant height and leaf dark green degree of flowering cabbage. Compared with Kairun, 25 wt% of pyraclostrobin nano-SC with the wetting agent had about 10% lower control efficiency against flowering cabbage downy mildew disease but showed no difference in plant height and leaf dark green degree of flowering cabbage. The effect of 25 wt% of pyraclostrobin nano-SC with the wetting agent can be comparable to that of Kairun. Moreover, nano-SC did not contain organic solvents, which is safer to store and transport, and also more environmentally friendly compared with EC. Therefore, nano-SC is expected to become the next generation of new environmentally friendly and high-efficiency pesticide formulations.

## 3. Materials and Methods

### 3.1. Materials

NaLS, provided by Fangzheng Chemical Auxiliary Factory (Tumen, Jilin, China), was obtained from the sulfite pulping spent liquor of poplar after purification and drying. Glycerin, cetyltrimethylammonium bromide (CTAB), ethanol and sodium dodecyl benzenesulfonate were all purchased from Guangzhou Chemical Reagent Co., Ltd. (Guangzhou, Guangdong, China). Pyraclostrobin was provided by Yongnong Bioscience Co., Ltd. (Shaoxing, Zhejiang, China), and the active ingredient content reached as high as 98%. Kairun, 25 wt% of pyraclostrobin emulsifiable concentrate (EC), was supplied by BASF Plant Protection Co., Ltd. (Nantong, Jiangsu, China). Commercial hyperbranched polycarboxylate D-3911, D-2912, D-2, straight-chain polycarboxylate SN-5040 and comb-type polycarboxylate TERSPERSE 2500 were provided by Dongguan Changzhou Chemical Engineering Co., Ltd. (Dongguan, Guangdong, China), Nopco Corp. (Kyoto, Japan) and Huntsman Corp., Salt Lake, UT, USA, respectively. Commercial lignin dispersant Reax 80D (the molecular weight and the sulfonic group content were 95,000 and 0.80 mmol/g, respectively) and Reax 85A (the molecular weight and the sulfonic group content were 10,000 and 0.80 mmol/g, respectively) were both purchased from MeadWestvaco Corp., Lansing, MI, USA.

### 3.2. Preparation of NaLS/CTAB Colloidal Spheres

NaLS/CTAB colloidal spheres were prepared via electrostatic and hydrophobic self-assembly based on our previous work [32]. Concretely speaking, 141 g of NaLS was dissolved in 94 g of deionized water, which was then stirred using a ZNCL-S-10D multiple-point magnetic heating plate (Yuhua Co., Ltd., Gongyi, Henan, China) at 800 rpm for 72 h to prepare 60 wt% of NaLS solution. In the same way, 50 g of CTAB was dissolved in an alcohol–water mixed solvent consisting of 30 g of ethanol and 20 g of water to prepare 50 wt% of CTAB solution. Next, a NaLS solution was mixed with a CTAB solution at a mass ratio of 2.35:1 to obtain a NaLS/CTAB mixing solution. Afterwards, 700 g of water was gradually added to the NaLS/CTAB solution under stirring within 30 min. After standing for 24 h, the formed precipitate was filtered and fully washed with water, followed by being processed through freeze drying. Finally, NaLS/CTAB colloidal spheres could be successfully obtained.

### 3.3. Characterizations of NaLS/CTAB Colloidal Spheres

The microstructure of NaLS/CTAB colloidal spheres was characterized by a field emission scanning electron microscope (SEM, SU8220, Hitachi Corp., Tokyo, Japan) and a transmission electron microscopy (TEM, JEM2100, Electronics Corp., Tokyo, Japan). Before SEM measurements, 3 μL of NaLS/CTAB colloidal dispersions with a mass concentration of 0.025% was dropped on the silicon wafer. After drying, this silicon wafer was adhered to the conductive adhesive, and then underwent a gold spraying treatment for tests. Next, the acceleration voltage of SEM was set at 10 kV, and the silicon wafer was photographed and recorded. Similarly, TEM samples were prepared by dropping 3 μL of 0.025 wt% of colloidal dispersions onto the copper grids, followed by being naturally dried at room temperature for measurements. Afterwards, the morphology of NaLS/CTAB colloidal spheres was observed by TEM, and the accelerated voltage was fixed at 80 kV.

### 3.4. Preparations of Pyraclostrobin Nano-SC

Pyraclostrobin nano-SC was prepared by a water-based grinding method based on the formula in Table S1. Specifically, pyraclostrobin, dispersants D-3911 and Reax 85A and the disperse medium (water) were separately weighted and added into an agate jar, and the pH value was then adjusted to 7 using citric acid, followed by adding zirconium dioxide balls (a diameter of 0.3 mm) as grinding materials to grind through a QM-3SP2 planetary ball mill (Miqi Instrument Co., Ltd., Changsha, China). Finally, 25 wt% of pyraclostrobin nano-SC could be successfully obtained.

### 3.5. Characterizations of Pyraclostrobin Nano-SC

The pyraclostrobin nano-SC D_90_ particle size was determined through a laser particle size analyzer (BetterSize 2600, Dandong Baite Instrument Co., Ltd., Dandong, Liaoning, China). An amount of 0.1 g of pyraclostrobin nano-SC was dropped into the sample tank for measurements at room temperature. At least three parallel measurements were carried out for each sample, and the average value was ultimately utilized. Then, according to the method in national standard [33], the prepared pyraclostrobin nano-SC was placed and sealed in a constant temperature drying oven (54 ± 1 °C) separately for 3 and 7 days. After being taken out, the nano-SC D_90_ particle size before and after thermal storage was also measured by the above laser particle size analyzer to estimate its thermal stability.

A 25 wt% of pyraclostrobin nano-SC was diluted 2000 times to observe its morphology through a SU8220 field emission SEM (Hitachi Corp., Tokyo, Japan) based on the above-mentioned same procedure with NaLS/CTAB colloidal spheres. The X-ray diffraction (XRD) spectrogram of original pyraclostrobin pesticide and pyraclostrobin nano-SC was determined on an X-ray diffractometer (D8 Advance, Bruker Corp., Kalka, German). Firstly, pyraclostrobin nano-SC was freeze-dried, and then measured using CuKα radiation as a light source at 50 mA and 60 kV in a 2θ wide-angle scanning range of 5–60° with a step size of 0.02°and a scanning speed of 6°/min. The rheological properties of pyraclostrobin nano-SC were tested by means of a rheometer (MARS Ⅲ, Thermo Fisher Scientific Co., Waltham, MA, USA) equipped with a CC26Ti rotor with a gap thickness of 1 mm at 25 °C. All measurements were conducted from 200 to 1 s^−1^ to obtain the viscosity/shear rate curve of nano-SC.

### 3.6. Farmland Experiments

In this section, 25 wt% of pyraclostrobin nano-SC, 25 wt% of pyraclostrobin SC and 25 wt% of pyraclostrobin EC (Kairun) were diluted by certain multiples for quality improvement and yield increase tests on the flowering cabbage with an average plant height of 35 cm to explore the prevention and treatment effect of these three preparations on flowering cabbage downy mildew disease. When the flowering cabbage was infected by this disease, yellow leaf phenomenon would appear. So, the number of yellow leaves could be used to estimate the control efficiency of pesticide preparations against flowering cabbage downy mildew disease. Using sodium dodecyl benzenesulfonate as a wetting agent, the quality improvement and yield enhancement effect of nano-SC and SC before and after adding the wetting agent was determined and also compared with EC. All measurements were carried out between 14:00 and 15:30 on 7 December 2022 in Nanning, Guangxi. During pesticide application, the temperature was kept at 16–24 °C and the relative humidity was about 60%. Table S2 provides the specific implementation scheme of the farmland experiments.

In this farmland experiment, an electric knapsack sprayer was used to spray stems and leaves, and it was thought to be appropriate to spray leaves wet without dripping water. The pesticide liquid amount applied was 30 L/mu (a frequently used measuring unit of land area in China, and 1 mu = 0.1647 acre) and the pesticide was applied once. After pesticide application for 3 days, the yellow leaf number, plant height, leaf color and growth of flowering cabbage were investigated. Taking one mu of farmland as a plot, 5 points were randomly selected from each plot, and 1 plant height was measured at each plot. Meanwhile, the number of yellow leaves in each treatment plot was counted.

## 4. Conclusions

In this work, 25 wt% of pyraclostrobin nano-SC was prepared through a water-based grinding method, and the best grinding formula was obtained and shown as follows: a grinding time of 23 h, D-3911 as a dispersant and a dispersant dosage of 12 wt%. The pyraclostrobin nano-SC D_90_ particle size prepared according to this best grinding condition was 215 nm, and nano-SC showed a near spherical shape. The XRD experimental results indicated that the main crystalline structures of pyraclostrobin in nano-SC were maintained after grinding. Then, the effects of glycerin as a stabilizer on the stability of pyraclostrobin nano-SC were investigated and it was found that, when nano-SC was placed at room temperature for 60 days, the delamination significantly improved with the increasing glycerin addition. But, after 3 days of thermal storage at 54 ± 1 °C, serious delamination and precipitation occurred in nano-SC, and the particle size also increased to the micron scale. To improve the thermal storage stability of pyraclostrobin nano-SC, colloidal spheres were prepared from a NaLS/CTAB mixing system at SMR via electrostatic and hydrophobic self-assembly. SEM and TEM observations showed that the microspheres were spherical in conformation, closely arranged and without agglomeration, and the particle sizes were all basically between 20 and 30 nm. When these prepared NaLS/CTAB colloidal spheres were added to pyraclostrobin nano-SC, the delamination and precipitation problems could be solved. In the meantime, the addition of colloidal spheres could make the nano-SC D_90_ particle size decrease from 2726 to 1023 nm after 7 days of thermal storage, indicating that NaLS/CTAB colloidal spheres could inhibit the particle size growth and improve the thermal storage stability of nano-SC. Finally, farmland experiments were conducted and the results showed the control efficiency of pyraclostrobin nano-SC against flowering cabbage downy mildew disease increased by about 30% compared with SC, and meanwhile the flowering cabbage leaves were darker green with a better growth, and the plant height increased by about 6–10%. After adding the wetting agent to pyraclostrobin nano-SC, there was basically no difference between nano-SC and the commercial Kairun (25 wt% of pyraclostrobin EC, currently the best pyraclostrobin formulation in the world) in terms of plant height and leaf color, and both had a comparable effect on the quality improvement and yield increase in flowering cabbage.

## Figures and Tables

**Table 1:** Quality improvement and yield increase effects of different pyraclostrobin preparations on flowering cabbage after application for 3 days.

Serial Number	Formulation	Dosage (g)	D_90_ Particle Size (Before Dilution) (nm)	Dosage of Wetting Agent (g)	Average Plant Height (cm)	Number of Yellow Leaves
1-1	25 wt% of pyraclostrobin SC	10	2985	0	44.0	18
1-2	25 wt% of pyraclostrobin SC	15	2985	0	45.6	20
2-1	25 wt% of pyraclostrobin nano-SC	10	215	0	46.8	12
2-2	25 wt% of pyraclostrobin nano-SC	15	215	0	46.2	10
3-1	25 wt% of pyraclostrobin SC	10	2985	0.5	44.2	15
3-2	25 wt% of pyraclostrobin SC	15	2985	0.5	44.4	20
4-1	25 wt% of pyraclostrobin nano-SC	10	215	0.5	47.8	6
4-2	25 wt% of pyraclostrobin nano-SC	15	215	0.5	48.4	7
5-1	25 wt% of pyraclostrobin EC (Kairun)	10	-	0	47.6	5
5-2	25 wt% of pyraclostrobin EC (Kairun)	15	-	0	49.8	5
CK	Water	15	-	0	42.8	25

## Data Availability

Data are contained within the article and Supplementary Materials.

## Supplementary Materials

The following supporting information can be downloaded at: https://www.mdpi.com/article/10.3390/molecules29071419/s1, Figure S1: SEM image of pyraclostrobin nano-SC with 10 wt% of glycerin after thermal storage for 7 days; Figure S2: Effects of different pyraclostrobin formulations on growth of flowering cabbage; Table S1: Grinding formula for preparing pyraclostrobin nano-SC; Table S2: Farmland experimental scheme of pyraclostrobin against cabbage downy mildew disease.
