# Supplementary material for: Preparations of 25 wt% of Pyraclostrobin Nanosuspension Concentrate (SC) Using Lignosulfonate-Based Colloidal Spheres to Improve Its Thermal Storage Stability
Source: Molecules. 2024 Mar 22;29(7):1419. doi: 10.3390/molecules29071419 (PMC11013202; doi:10.3390/molecules29071419)
Supplement: Supplementary file 1 [file molecules-29-01419-s001.zip › molecules-2905028-supplementary.pdf]

Supplementary Materials

Preparations of 25 wt% of Pyraclostrobin Nanosuspension Concentrate (SC) Using Lignosulfonate-Based Colloidal Spheres to Improve Its Thermal Storage Stability

Qianqian Tang [2], Yu Sun [1], Jinnuo Li [2], Mingsong Zhou [1,*], Dongjie Yang [1] and Yuxia Pang [1]

[1] State Key Laboratory of Pulp and Paper Engineering, School of Chemistry and Chemical Engineering, South China University of Technology, 381 Wushan Road, Tianhe District, Guangzhou 510640, China; lscxcyjy@163.com (Y.S.); cedjyang@scut.edu.cn (D.Y.); ceyxpang@scut.edu.cn (Y.P.)

[2] Henan Key Laboratory of Function-Oriented Porous Materials, College of Chemistry and Chemical Engineering, Luoyang Normal University, 6 Jiqing Road, Yibin District, Luoyang 471934, China; lhltqq1987@163.com (Q.T.); 17333740259@163.com (J.L.)

* Correspondence: mszhou@scut.edu.cn (M.Z.); Tel.: +86-20-87114722

Number of Pages: 5
Number of Figures: 2
Number of Tables: 2

Figure S1. SEM image of pyraclostrobin nano-SC with 10 wt% of glycerin after thermal storage for 7 days.

Figure S2. Effects of different pyraclostrobin formulations on growth of flowering cabbage.

Table S1. Grinding formula for preparing pyraclostrobin nano-SC.

Table S2. Farmland experimental scheme of pyraclostrobin against cabbage downy mildew disease.

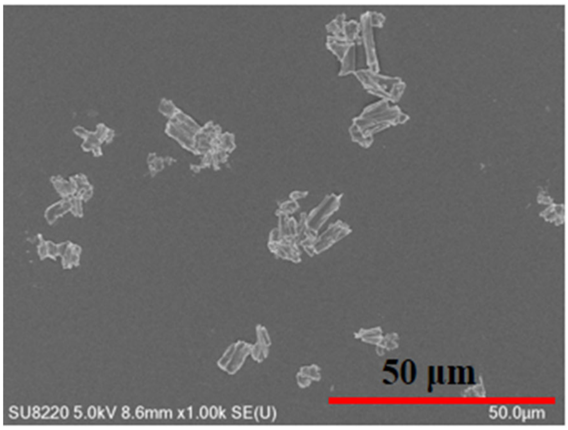

Figure S1. SEM image of pyraclostrobin nano-SC with 10 wt% of glycerin after thermal storage for 7 days.

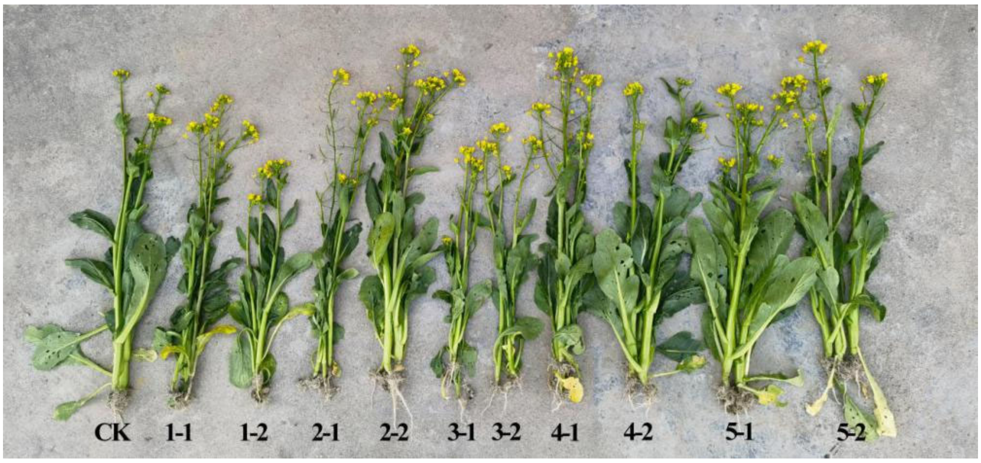

Figure S2. Effects of different pyraclostrobin formulations on growth of flowering cabbage.

Table S1. Grinding formula for preparing pyraclostrobin nano-SC.

Components	Raw materials	Dosage (wt%)
Pesticide	Pyraclostrobin	25
Dispersant	Lignin dispersants Reax 80D, Reax 85A, polycarboxylate D-3911, D-2912, D-2, SN-5040, TERSPERSE 2500	12
pH controlling agent	Citric acid	A small amount
Disperse medium	Deionized water	Added to 100%
Total		100

Table S2. Farmland experimental scheme of pyraclostrobin against cabbage downy mildew disease.

Serial number	Formulation	Dilution times	Dosage of formulation (g)	Dosage of wetting agent (g)	D_{90} particle size (before dilution) (nm)
1-1	25 wt% of pyraclostrobin SC	750	10	0	2985
1-2	25 wt% of pyraclostrobin SC	500	15	0	2985
2-1	25 wt% of pyraclostrobin nano-SC	750	10	0	215
2-2	25 wt% of pyraclostrobin nano-SC	500	15	0	215
3-1	25 wt% of pyraclostrobin SC	750	10	0.5	2985
3-2	25 wt% of pyraclostrobin SC	500	15	0.5	2985
4-1	25 wt% of pyraclostrobin nano-SC	750	10	0.5	215
4-2	25 wt% of pyraclostrobin nano-SC	500	15	0.5	215
5-1	25 wt% of pyraclostrobin EC (Kairun)	750	10	0	-
5-2	25 wt% of pyraclostrobin EC (Kairun)	500	15	0	-
CK	Water	-	15	0	-
